# Electrical stimulation of the cochlea for treatment of chronic disabling tinnitus: an open-label trial towards the development of an implantable device

**DOI:** 10.1186/s12967-022-03271-4

**Published:** 2022-01-29

**Authors:** John P. Marinelli, C. Lane Anzalone, Christoph M. Prummer, Gayla L. Poling, Jeffrey P. Staab, Nicole M. Tombers, Christine M. Lohse, Matthew L. Carlson

**Affiliations:** 1 Department of Otolaryngology-Head and Neck Surgery, Mayo Clinic, Rochester, MN 200 1st St SW55905 USA; 2 Department of Otolaryngology-Head and Neck Surgery, San Antonio Uniformed Services Health Education Consortium, JBSA, Oddly, TX USA; 3 Department of Psychiatry and Psychology, Mayo Clinic, Rochester, MN USA; 4 Department of Quantitative Health Sciences, Mayo Clinic, Rochester, MN USA; 5 Department of Neurologic Surgery, Mayo Clinic, Rochester, MN USA

**Keywords:** Tinnitus, Treatment, Electrical stimulation, Promontory stimulation, Implantable device, Cochlear implant, Clinical trial

## Abstract

**Background:**

Chronic tinnitus affects millions of people globally and constitutes the most commonly compensated disability among military service members in the United States. Existing treatment options largely surround helping patients cope with their disease as opposed to directly suppressing tinnitus perception. The current study investigated the efficacy of electrical stimulation of the cochlea on chronic disabling tinnitus.

**Methods:**

In this single-arm, open-label clinical trial, 22 adult subjects with severe-range asymmetric or unilateral non-pulsatile tinnitus underwent electrical stimulation of the cochlea through use of an extra-cochlear electrode positioned on the cochlear promontory. Each subject underwent 3 stimulation treatments over 3 weeks at 7-day intervals. Tinnitus severity was determined by Tinnitus Handicap Inventory (THI), Tinnitus Functional Index (TFI), and Tinnitus Visual Analog Scale (VAS). Inclusion criteria required subjects have no worse than moderate sensorineural hearing loss determined by pre-enrollment audiometric testing. The primary outcome was nadir post-treatment THI scores, obtained at seven timepoints following electrical stimulation, with clinically significant improvement defined as a decrease of  ≥ 7.

**Results:**

All 22 (100%) subjects experienced clinically significant improvement in the THI during the study period with a mean decrease in scores of − 31 (95% CI − 38 to − 25) from a baseline of 48. Twenty (91%) experienced clinically significant improvement detectable on at least two of the three tinnitus survey instruments and 17 (77%) experienced clinically significant improvement detectable on all three survey instruments (i.e., THI, TFI, and VAS). Eight (36%) subjects reported either complete (THI of 0; n  = 3) or near-complete (THI 1–4; n  = 5) suppression of their tinnitus following a stimulation session. Thirteen (59%) subjects reported a nadir following stimulation at or below the threshold for “no or slight handicap” on the THI (≤ 16). No adverse events were observed.

**Conclusions:**

These findings establish the foundation for the development of an extra-cochlear implantable device that delivers electrical stimulation to the cochlea for the treatment of disabling tinnitus. For patients considering device implantation, trans-tympanic cochlear promontory stimulation can facilitate patient selection.

*Trial Registration* ClinicalTrials.gov Identifier: NCT03759834. URL: https://clinicaltrials.gov/ct2/show/NCT03759834

**Supplementary Information:**

The online version contains supplementary material available at 10.1186/s12967-022-03271-4.

## Background

In the absence of approved pharmacological or surgical therapies, millions of people live with disabling chronic tinnitus [1–3]. It is estimated that 2 million residents of the United States are severely impacted by chronic tinnitus, and tinnitus constitutes the most commonly compensated service-connected disability among veterans nationally [1–3]. Although the etiology of tinnitus is complex and multifactorial, [3] growing evidence surrounding tinnitus outcomes among patients undergoing cochlear implantation for moderate to profound sensorineural hearing loss has demonstrated that almost 90% of patients with preoperative tinnitus experience notable improvement and up to 45% experience complete suppression of their tinnitus during device use [4–7].

Analogous to the nerve stimulation treatment of neuropathic pain in peripheral nerve disorders, the therapeutic effect of cochlear implantation on chronic tinnitus appears to be related to electrical stimulation of the cochlea and independent of auditory masking [8]. Moreover, the observed therapeutic effect has been shown to be durable up to at least 10 years [9]. Yet, because cochlear implantation places native hearing at risk, it is contraindicated in patients with functional natural hearing. However, most patients with disabling chronic tinnitus have no worse than moderate hearing loss [1, 3]. Attempting to replicate the electrical stimulation of the cochlea during cochlear implant device use, an open label clinical trial was undertaken to evaluate the utility of trans-tympanic electrical stimulation of the cochlear promontory in adults who suffer from bothersome chronic unilateral or asymmetric tinnitus but do not have hearing loss of sufficient severity to qualify for cochlear implantation.

## Methods

After obtaining institutional review board approval, the protocol was registered with ClinicalTrials.gov (NCT03759834). Enrollment criteria stipulated that subjects were  ≥ 18 years old with no worse than moderate sensorineural hearing loss in the study ear (based on a pure-tone average of hearing thresholds at 500, 1000, 2000 Hz, and 3000 Hz of  < 70 dB on pre-enrollment audiometric testing). Word recognition scores had to be  > 75%. Inclusion criteria required subjects undergo pre-enrollment neuroimaging with both gadolinium-enhanced magnetic resonance imaging of the head and high-resolution computed tomography of the temporal bones, and both studies had to be read as normal for subject age by a neuroradiologist from our institution.

Subjects’ tinnitus had to be non-pulsatile, unilateral or definitively asymmetric by subject report, and be present for at least 6 months but less than 3 years. Subjects’ tinnitus had to be disruptive, determined by subject reporting “severe-range” tinnitus on the Tinnitus Handicap Inventory (THI), [10, 11] Tinnitus Functional Index (TFI), [12] or Tinnitus Visual Analog Scale (VAS) [13]. The tinnitus also had to be intractable and fail conventional methods of therapy such as auditory masking with hearing aid use.

Exclusion criteria excluded subjects who were pregnant, had a history of brain or lateral skull base surgery, had a history of major head trauma, and those actively using any tinnitus treatments outside of masking devices (e.g., noise generators, hearing aids). Subjects could not be taking antidepressants, anxiolytics, or antipsychotics and could not have clinically significant anxiety (determined by a pre-enrollment Generalized Anxiety Disorder-7 score  > 9), clinically significant depression (determined by a pre-enrollment Patient Health Questionnaire-8 score  > 9), or hypochondriacal level illness anxiety (determined by a pre-enrollment Short Health Anxiety Inventory score  > 25). Subjects received no financial compensation for participation. The study was performed between December 2017 and September 2018.

**Fig. 1:**
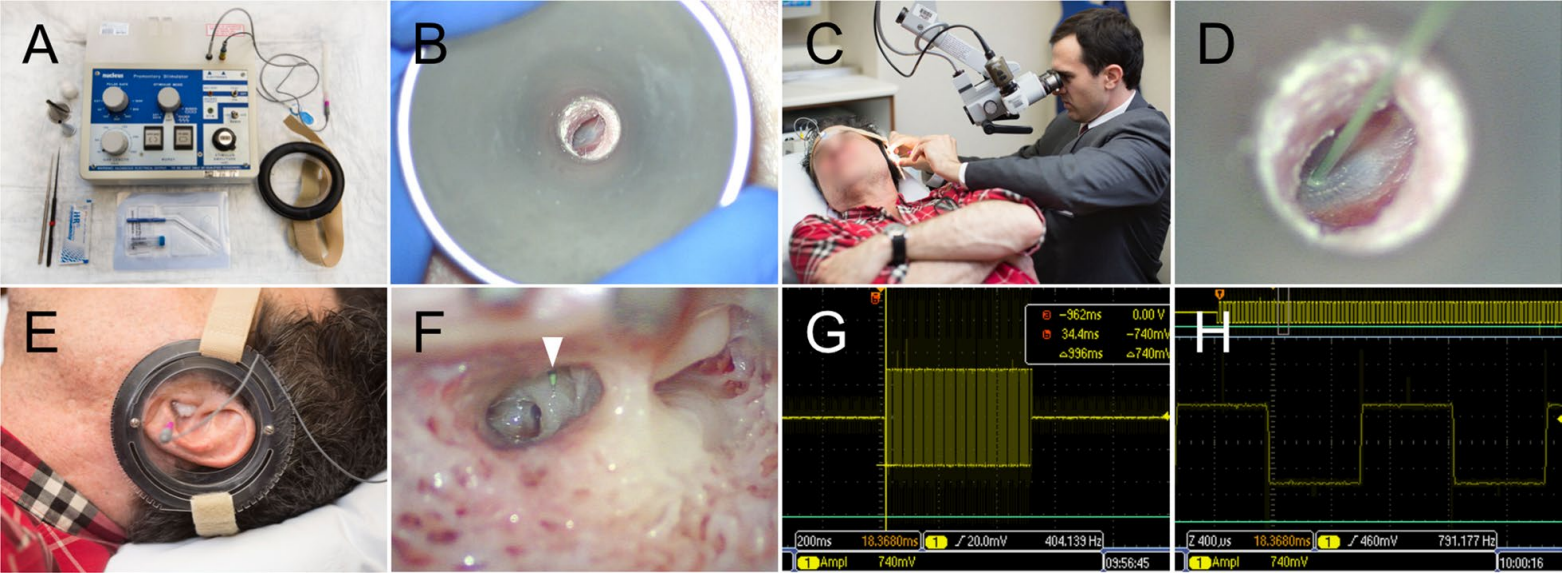
Trans-tympanic electrical stimulation of the cochlea. **A** Equipment used for promontory stimulation, including the Cochlear Nucleus Promontory Stimulator Z10012^®^ (Cochlear Corporation, Melbourne, Australia). **B** Otomicroscopic view of the left tympanic membrane. **C**, **D** Surgeon placing the trans-tympanic monopolar needle electrode on the promontory. **E** Stabilized electrode in left ear. **F** Typical location of the needle electrode on the promontory of the cochlea is shown (white arrowhead) through a facial-recess approach commonly used for cochlear implantation[22]. **G**, **H** oscilloscope recording of promontory stimulation output demonstrating charge-balanced square biphasic pulse waveforms

Each subject underwent 3 stimulation treatments over 3 weeks at 7-day intervals. Promontory stimulation was performed using biphasic charge balanced pulses (Cochlear Nucleus Promontory Stimulator Z10012^®^, Cochlear Corporation, Melbourne, Australia; Fig. 1). Following trans-tympanic placement of an insulated monopolar stimulation probe, electrode impedance testing was performed to evaluate proper electrode placement. Gentle repositioning of the electrode or replacement through the tympanic membrane was performed when impedance values were not acceptable. Next, calibration testing to assess optimal stimulation parameters for each therapeutic session was carried out using an output of 0–1000 µA for pulse frequencies 100, 800, and 1600 Hz (Additional file 1: Table S1). As an internal control function, testing included a run of “on–off” stimulations blinded to the subject (i.e., subject was blinded to the status of the “on” or “off” position of the stimulation machine) to document subject perception of stimulation. Electrical current levels were gradually titrated to determine the maximal comfortable threshold as reported by the subject. The subject then underwent 10 min of trans-tympanic electrical stimulation at 80% of the maximum comfort threshold for each of the predetermined pulse frequencies. The primary outcome measure of interest was nadir in THI following electrical stimulation.

Immediately prior to each treatment, comprehensive audiometric testing was performed. To monitor the potential for delayed audiologic complications, physical examination with binocular microscopy and comprehensive audiometric testing was completed at 3 months following the third promontory stimulation treatment. Information regarding the specific tinnitus survey instruments, subject survey reporting schedule, and statistical power estimations can be accessed in the online supplement (Additional file 1: Text S1).

## Results

Twenty-five subjects initially enrolled in the study. Three subjects withdrew prior to undergoing promontory stimulation due to an inability to complete the multiple requirements of the study. Therefore, 22 subjects underwent promontory stimulation (Table 1); 21 (95%) subjects completed the study, with one subject withdrawing after the first stimulation treatment due to a perceived inability to complete the remaining requirements in the study; their reported data until self-exclusion were included in efficacy and safety endpoints. No subjects experienced any adverse events, including no persistent pain or discomfort following stimulation and no subjective change in hearing thresholds. Further, physical exam and comprehensive audiometric testing demonstrated no clinically detectable change from baseline to the 3-month post-treatment testing (Additional file 1: Table S2).

**Table 1:** Baseline clinical features of study cohort, n  = 22

Feature^a^	
Demographics	
Age at enrollment in years	59 (8)
Sex	
Female	8 (36)
Male	14 (64)
Eligibility criteria	
Tinnitus Handicap Inventory	57 (16)
Tinnitus Functional Index	70 (10)
Visual Analog Scale	7.9 (1.4)
Generalized Anxiety Disorder-7	1.5 (0–3)
Patient Health Questionnaire-8	2.5 (1–5)
Short Health Anxiety Inventory	7.5 (5.6)
Tinnitus characteristics	
Duration of tinnitus in years (*n* = 19)	2.0 (0.8)
Persistence (*n* = 20)	
Constant	18 (90)
Intermittent	2 (10)
Fluctuations in intensity or loudness (*n* = 20)	15 (75)
Treatments (*n* = 20)	
Masker	8 (40)
Hearing aid	5 (25)
Music therapy	3 (15)
Tinnitus retraining therapy	1 (5)
Counseling	1 (5)
None	5 (25)

^a^Summarized with mean (SD), median (IQR), or n (%)

All 22 (100%) subjects experienced clinically significant improvement in the THI (score decrease by  ≥ 7) [11] during the study period with a mean decrease in scores of − 31 (95% CI − 38 to − 25) from a baseline of 48 (Table 2). Twenty (91%) experienced clinically significant improvement detectable on at least two of the three tinnitus survey instruments and 17 (77%) experienced clinically significant improvement detectable on all three survey instruments (i.e., THI, TFI, and VAS). Eight (36%) subjects reported either complete (THI of 0; n  = 3) or near-complete (THI 1–4; n  = 5) suppression of their tinnitus following a stimulation session. Thirteen (59%) subjects reported a nadir following stimulation at or below the threshold for “no or slight handicap” on the THI (≤ 16). At 3 months, most subjects had returned close to their baseline tinnitus severity levels, although 4 (18%) subjects exhibited sustained suppression of  ≤ 16 on the THI.

**Table 2:** Tinnitus severity survey reporting during study duration, n  = 22

Survey	Baseline^a^	Post-stimulation Session Nadir^a^	Difference^a,b^	95% CI for difference
THI	48 (15)	17 (17)	− 31 (14)	− 38 to − 25
TFI	60 (13)	25 (20)	− 35 (16)	− 42 to − 28
VAS	7.1 (1.4)	3.4 (2.5)	− 3.7 (2.1)	− 4.7 to − 2.8

*THI *Tinnitus Handicap Inventory; *TFI *Tinnitus Functional Index; *VAS *Visual Analog Scale

^a^Summarized with mean (SD)

^b^Defined as lowest post-stimulation score minus baseline score. Baseline scores were defined as the mean of the enrollment, baseline evaluation 1, baseline evaluation 2, baseline evaluation 3, and pre-stimulation 1 scores. Post-stimulation session nadir scores were defined as the lowest score reported following a stimulation session

## Discussion

Cochlear implantation has emerged as an effective treatment for bothersome chronic tinnitus in adults with concomitant moderate to profound sensorineural hearing loss [5, 7]. The therapeutic effect appears independent of the improved access to sound and has been shown to be durable in long-term follow-up [8, 9]. Preliminary mechanistic evidence suggests that the electrical current levels delivered by most modern cochlear implants may inactivate voltage-gated calcium channels that play a role in tinnitus generation [14]. Although cochlear implantation is contraindicated in patients with functional natural hearing, the current clinical trial demonstrates that therapeutic electrical stimulation of the cochlea is replicable for temporary periods through trans-tympanic cochlear promontory stimulation.

In the current clinical trial, the mean effect size of subjects’ nadir tinnitus level post-treatment exceeded the threshold for clinically significant decreases on the THI by over four-fold. This finding is notable because study subjects’ baseline THI is similar to a large proportion of patients suffering from chronic severe tinnitus, and the THI has been widely validated across multiple international studies [11, 15–19]. The clinical significance of the treatment response observed in the present work is also strengthened by a United States survey of 439 civilians and 269 military service members where nearly 75% of respondents reported being willing to receive an implantable device if it reduced tinnitus by even 50% [20]. Of note, the rate of complete or near-complete suppression of subjects’ tinnitus following trans-tympanic promontory stimulation (approximately 40%) is comparable to prior rates observed in the setting of cochlear implantation [7]. Notwithstanding the improvement in tinnitus severity among all study subjects, the duration of tinnitus suppression observed following trans-tympanic cochlear promontory stimulation was limited and returned close to baseline by 3 months following the last stimulation session for most subjects.

The notable but temporary tinnitus suppression effect of trans-tympanic electrical stimulation of the cochlea establishes a foundation for the development of an implantable device for treatment of disabling chronic tinnitus. An implantable device that does not place native hearing at risk would enable patients with functional natural hearing to potentially experience suppression of tinnitus on a daily basis similar to cochlear implant recipients. In this way, an implantable device would not be limited to brief stimulation sessions, as in trans-tympanic promontory stimulation, but would provide extended durations of tinnitus suppression based on current battery life capabilities of modern cochlear implant technology. Moreover, because one of the most common complaints among those suffering from tinnitus is lack of control of symptoms, a device that affords patients the ability to turn on/off the therapeutic benefit would likely confer unique psychological benefit in a patient population that often suffers from concomitant anxiety and depression [1, 12].

Important when considering an implantable device, several pieces of evidence suggest that it is unlikely that long-term electrical stimulation of the cochlea by an extra-cochlear electrode that does not violate the cochlear lumen would negatively influence patients’ baseline hearing. One of the strongest supports for this extends from the cochlear implant literature. For instance, Roland et al. evaluated long-term audiometric outcomes for patients with high-frequency sensorineural hearing loss who underwent cochlear implantation with the Nucleus^®^ HybridTM L24 (Cochlear Ltd., Sydney, Australia) cochlear implant and had at least 5 years of post-implantation follow-up [21]. The authors found that hearing changes from preoperative to the early postoperative timeframe were significant, but changes 6 months through 5 years post-activation of cochlear stimulation with a cochlear implant were not statistically significantly different [21]. They, along with other similar studies [22], demonstrated that the risk of sensorineural hearing loss following cochlear implantation is due to the upfront risk of violating the cochlear lumen, but the risk of progressive hearing loss after surgery mirrors the slow decline in hearing seen in the opposite, non-implanted ear. In other words, isolated chronic electrical stimulation of the cochlea does not appear to accelerate hearing loss compared to the non-stimulated ear. Similar durable hearing thresholds following long-term electrical stimulation of the cochlea using extra-cochlear electrodes have been demonstrated in animal models [23, 24]. Taken together, there is good reason to believe that extra-cochlear electrical stimulation of the cochlea in humans is unlikely to be harmful to hearing thresholds long-term.

Because the etiology of tinnitus is broad and response to therapy—to include cochlear implantation—varies among patients, patient selection for an extra-cochlear implantable device can be facilitated by a pre-implantation trial of trans-tympanic cochlear promontory stimulation. Twelve subjects who experienced clinically significant improvement across all three tinnitus survey instruments in the current study self-identified as desiring to undergo device implantation with an extra-cochlear implantable device based on existing cochlear implant technology that is currently under investigation (ClinicalTrials.gov number NCT03988699). Taken together, the current study demonstrates the feasibility and need for an implantable device that replicates the electrical stimulation of the cochlea during cochlear implant device use but does not risk patients’ native hearing.

## Conclusions

Electrical stimulation of the cochlea through a trans-tympanically placed electrode can suppress tinnitus. These results support the possibility that an extra-cochlear implantable device to deliver electrical stimulation to the cochlea can replicate the tinnitus suppression benefits of cochlear implants for most patients with chronic tinnitus who do not have hearing loss of sufficient severity to qualify for cochlear implantation. Given the results of the present study, trans-tympanic cochlear promontory stimulation may facilitate patient selection for device implantation.

## Supplementary Information

**Additional file 1: Table S1.** Summary of promontory stimulation parameters, n  = 22. **Table S2.** Safety data from behavioral audiometric testing across study duration, n  = 22. **Text S1.** Supplementary methods of tinnitus survey information and reporting schedule and statistical power.

## Data Availability

Not applicable.

## Abbreviations

THI: Tinnitus Handicap Inventory
TFI: Tinnitus Functional Index
VAS: Tinnitus Visual Analog Scale
