# Supplementary material for: Electrical stimulation of the cochlea for treatment of chronic disabling tinnitus: an open-label trial towards the development of an implantable device
Source: J Transl Med. 2022 Jan 29;20:56. doi: 10.1186/s12967-022-03271-4 (PMC8800213; doi:10.1186/s12967-022-03271-4)
Supplement: Supplementary file 1 — Additional file 1: Table S1. Summary of promontory stimulation parameters, n = 22. Table S2. Safety data from behavioral audiometric testing across study duration, n = 22. Text S1. Supplementary methods of tinnitus survey information and reporting schedule and statistical power. [file 12967_2022_3271_MOESM1_ESM.docx]

**Additional file**

Table of Contents

Table S1. Summary of promontory stimulation parameters………...………...……....……………………………….2

Text S1. Supplementary methods of tinnitus survey information and reporting schedule and statistical power 3

Table S2. Safety data from behavioral audiometric testing across study duration 4

References 5

**Table S1. Summary of promontory stimulation parameters, N=22.**

| **Feature*** |  |
| --- | --- |
|  |  |
| *First Session* |  |
| 100Hz |  |
| Minimum threshold | 40 (24-68) |
| First discomfort level | 98 (67-180) |
| 80% discomfort level | 85 (60-144) |
| 800Hz (*N=21*) |  |
| Minimum threshold | 130 (105-173) |
| First discomfort level | 240 (166-390) |
| 80% discomfort level | 192 (133-312) |
| 1600Hz |  |
| Minimum threshold | 213 (155-280) |
| First discomfort level | 354 (240-490) |
| 80% discomfort level | 286 (192-392) |
|  |  |
| *Second Session* (*N=21*) |  |
| 100Hz |  |
| Minimum threshold | 40 (25-78) |
| First discomfort level | 115 (100-160) |
| 80% discomfort level | 92 (80-128) |
| 800Hz |  |
| Minimum threshold | 160 (142-180) |
| First discomfort level | 262 (220-376) |
| 80% discomfort level | 210 (170-301) |
| 1600Hz |  |
| Minimum threshold | 240 (225-260) |
| First discomfort level | 350 (275-588) |
| 80% discomfort level | 280 (220-470) |
|  |  |
| *Third Session* (*N=21*) |  |
| 100Hz |  |
| Minimum threshold | 44 (22-90) |
| First discomfort level | 115 (86-184) |
| 80% discomfort level | 92 (69-147) |
| 800Hz |  |
| Minimum threshold | 150 (110-215) |
| First discomfort level | 310 (220-460) |
| 80% discomfort level | 248 (176-368) |
| 1600Hz |  |
| Minimum threshold | 270 (190-315) |
| First discomfort level | 416 (310-650) |
| 80% discomfort level | 333 (248-520) |
|  |  |
| *Summarized with median (IQR). Units of stimulation are in µA. | |

**Text S1. Supplementary methods of tinnitus survey information and reporting schedule and statistical power.**

***Tinnitus Survey Information and Reporting Schedule***

The Tinnitus Handicap Inventory (THI) was developed in 1996 and has been widely validated in multiple languages.^1-7^ For the purposes of the current investigation, a score cutoff of ≥56/100 is used to designate severe tinnitus handicap for study inclusion, and a change of ≥7 points is considered clinically significant. The Tinnitus Functional Index (TFI) was developed by Meikle, et al. in 2012.^8^ For the purposes of the current investigation, a score cutoff of ≥52/100 is used to designate severe tinnitus for study inclusion, and a change of ≥13 points is considered clinically significant. The Tinnitus Visual Analog Scale-A (VAS-A) and Tinnitus VAS-L are self-reported psychometric measuring instruments designed to determine severity of tinnitus annoyance and severity of tinnitus loudness, respectively. The VAS-A and VAS-L instruments were developed by Adamchic et al. in 2012.^9^ For the purposes of the current investigation, the VAS-A was used, and inclusion criteria required subjects score ≥5 out of 10. A change in score of ≥2 points are considered clinically significant. All surveys are self-reported by the patient. For inclusion, patients had to meet the severity level in at least one of the three instruments. In the current study, 10/22 subjects met criteria in all three, and the remaining met criteria in 2 of 3.

Following baseline surveying, THI, TFI, and Tinnitus VAS questionnaires were administered immediately prior to each stimulation session, during the last 10 minutes of promontory stimulation, 10 minutes following promontory stimulation, 1 hour after treatment, 24 hours after treatment, 48 hours after treatment, and 1 week after treatment. During each week post-treatment, subjects also recorded survey data at a point where they felt their tinnitus was maximally suppressed. At the 3-month follow up visit, these questionnaires were completed once again.

***Statistical Power***

Assuming a statistical power of 80% and a one-sided significance level of 0.05, a sample size calculation was performed to determine the number of subjects needed to detect a clinically significant change in tinnitus based on the THI. The calculation suggested that if the observed mean change in THI was 15 points with a standard deviation of 15, a change of ≥7 points could be detected with 24 subjects.

**Table S2. Safety data from behavioral audiometric testing across study duration, N=22.**

| **Hz (dB HL)** | **Pre-stim 1*** | **Most Recent*** | **Difference*^†^** | **95% CI for Difference** |
| --- | --- | --- | --- | --- |
| 250 | 8 (12) | 6 (12) | −2 (8) | −5 to 2 |
| 500 | 18 (12) | 16 (11) | −2 (9) | −6 to 2 |
| 1000 | 12 (15) | 11 (11) | −1 (9) | −5 to 3 |
| 1500^‡^ | 24 (14) | 21 (11) | −3 (5) | −7 to 0 |
| 2000 | 24 (14) | 23 (15) | −1 (6) | −4 to 1 |
| 3000 | 32 (18) | 30 (18) | −2 (5) | −4 to 0 |
| 4000 | 34 (20) | 32 (22) | −2 (5) | −4 to 0 |
|  |  |  |  |  |
| **WRS (%)^‡^** | 96 (8) | 95 (7) | −1 (6) | −4 to 2 |
|  |  |  |  |  |
| **Tympanometry^§^** |  |  |  |  |
| A | 17 (77) | 17 (77) |  |  |
| As | 3 (14) | 2 (9) |  |  |
| Ad | 2 (9) | 1 (5) |  |  |
| B | 0 | 2 (9) |  |  |
|  |  |  |  |  |
| *Summarized with mean (SD) or n (%).  ^†^Defined as most recent minus pre-stim 1.  ^‡^N=11 for 1500 Hz; N=18 for WRS.  ^§^16 subjects remained A, 2 remained As, 1 remained Ad, 1 switched from A to B, 1 switched from Ad to B, and 1 switched from As to A. No subjects had persistent perforations in the tympanic membrane at 3 months. | | | | |

**References**

1. Limviriyakul S, Supavanich W. The validity and reliability of tinnitus handicap inventory Thai version. J Med Assoc Thai 2012;95:1433-40.

2. Barake R, Rizk SA, Ziade G, Zaytoun G, Bassim M. Adaptation of the Arabic Version of the Tinnitus Handicap Inventory. Otolaryngol Head Neck Surg 2016;154:508-12.

3. Tobias CA, Llanes EG, Chiong C. Validity of a filipino translation of the Tinnitus Handicap Inventory. Int Tinnitus J 2012;17:64-9.

4. Monzani D, Genovese E, Marrara A, et al. Validity of the Italian adaptation of the Tinnitus Handicap Inventory; focus on quality of life and psychological distress in tinnitus-sufferers. Acta Otorhinolaryngol Ital 2008;28:126-34.

5. Zachariae R, Mirz F, Johansen LV, Andersen SE, Bjerring P, Pedersen CB. Reliability and validity of a Danish adaptation of the Tinnitus Handicap Inventory. Scand Audiol 2000;29:37-43.

6. Newman CW, Jacobson GP, Spitzer JB. Development of the Tinnitus Handicap Inventory. Arch Otolaryngol Head Neck Surg 1996;122:143-8.

7. Zeman F, Koller M, Figueiredo R, et al. Tinnitus handicap inventory for evaluating treatment effects: which changes are clinically relevant? Otolaryngol Head Neck Surg 2011;145:282-7.

8. Meikle MB, Henry JA, Griest SE, et al. The tinnitus functional index: development of a new clinical measure for chronic, intrusive tinnitus. Ear Hear 2012;33:153-76.

9. Adamchic I, Langguth B, Hauptmann C, Tass PA. Psychometric evaluation of visual analog scale for the assessment of chronic tinnitus. Am J Audiol 2012;21:215-25.
